# Evolution of Bacterial Cellulose in Cosmetic Applications: An Updated Systematic Review

**DOI:** 10.3390/molecules27238341

**Published:** 2022-11-30

**Authors:** Thais Jardim Oliveira, Talita Cristina Mena Segato, Gabriel Pereira Machado, Denise Grotto, Angela Faustino Jozala

**Affiliations:** 1 LAMINFE—Laboratory of Industrial Microbiology and Fermentation Process, University of Sorocaba, Sorocaba 18023-000, SP, Brazil; 2 LAPETOX—Laboratory of Toxicological Research, University of Sorocaba, Sorocaba 18023-000, SP, Brazil

**Keywords:** bacterial cellulose, cell culture, cosmetics, microbiology, polymers, skincare

## Abstract

In recent decades, there has been an increase in environmental problems caused by cosmetic products derived from toxic substances. Based on this issue, researchers and developers of new beauty cosmetics are looking for new natural alternatives that work well for the consumer and have biodegradable characteristics. This systematic review highlights the major publications of bacterial cellulose used strictly for cosmetics in the last 10 years. Bacterial cellulose is a natural product with great cosmetic properties and low cost that has shown excellent results. This study aimed at collecting rigorous information on bacterial cellulose in the cosmetic field in the last decade to produce a systematized review. A comprehensive search was conducted with selected descriptors involving the topic of “bacterial cellulose”, “cosmetics”, “clean beauty”, and “skin mask”. Seventy studies were found, which went through exclusion criteria that selected only those related to the topic that was searched. In the 12 remaining studies that met the criteria, bacterial cellulose showed conditions for use as a mask-forming product for facial care. The increase in the number of publications concerning bacterial cellulose in cosmetics in the last ten years is a strong indicator that this is a growing area for both research and the industry.

## 1. Introduction

There has been an increase in the environmental contamination caused by cosmetic and personal care products produced from raw materials with toxic properties and also substances derived from petroleum [1]. The substances used in cosmetics and personal care product are safe for consumers, but most of the time they are harmful to the environment. They can create problems when they are incorrectly discarded [2], or after use, when they go into water that eventually returns to the environment through effluents, as is the case with synthetic microbeads [3].

Some formulations use petroleum-derived substances as raw material, which end up resulting in bioaccumulation and biomagnification in aquatic species, causing a significant imbalance in their environment [2]. The long persistence of organic pollutants is also responsible for diseases such as allergy, endocrine and microbiota disorders, and cancer. Parabens, phthalates, toluene, formaldehyde, and sulfates are examples of toxic substances that may lead to these kinds of diseases. These substances interfere in the hormonal system, generating toxicity in the organism, causing imbalances in human health and the environment [1,4].

In this context, the need to seek new technologies arises in order to meet the demand of both final consumers, with efficient dermatological properties [5], and the ecosystem, without presenting toxicity [2]. With this movement of cosmetic and personal care industries in the search for products that are safe and ecofriendly, the application of bacterial cellulose as a natural vehicle for dermatological alternatives is highly appreciated [6].

Bacterial cellulose (BC) is a polymer, considered an ecofriendly biomaterial since it does not cause ecological pollution [7], and it also has a biodegradable nature [8]. As a cosmetic, BC is particularly promising with several dermo-pharmacological applications due to its biological properties, such as: morphology, which has organized arrangement of nanofibers, facilitating the transport and delivery of substances, without interfering in the product function [7,9]; high mechanical strength; purity; high water absorption; no toxic characteristics; and most importantly, high biocompatibility. In addition, it can replace synthetic, petroleum-derived and toxic-ecological cosmetic ingredients [6].

The production of BC is carried out through the synthesis of extracellular polysaccharides produced by some non-pathogenic bacterial strains. This polysaccharide is formed by fibers and nanofibers that form around the bacterial cells [10].

Considering skin care cosmetics, BC as a natural biodegradable polymer has proven to be a very versatile product [9,11] once its characteristics are appropriate for this type of application. Moreover, BC has been studied as a potential substitute for synthetic polymers that are commonly used by the cosmetics industry, such as polyacrylamide, nylon, and polyethylene, compounds whose toxicities are widely known and studied for replacement [12].

Also in cosmetic application, BC has been noticed as an excellent system for delivering compounds to the skin due to its high adherence and water absorption [6]. In a study reported in 2020, vitamin B was incorporated into BC membranes and its distribution property was tested. The results proved that there was release of vitamin B, and the in vitro tests showed no cytotoxicity, indicating that this type of BC-based product is suitable and safe for skin care [11,12].

Recently, in 2021, BC was evaluated as a delivery system for *Epilobium angustifolium* extract, a natural antioxidant. The natural compound was loaded into BC membranes and tested in fibroblast cells and in ex vivo pig skin, via a cell permeator. The results indicated that the delivery of the antioxidant was accomplished, and the system showed no cytotoxicity. In that study, BC membranes were indicated as a promising solution for the use of topical antioxidants [10].

When the environmental positive impact is added to the promising characteristics of the BC uses in cosmetics, the increase in the value chain for this biopolymer is remarkable [6,12]. Therefore, this review aimed systematically to highlight studies that contextualize the evolution of the research and development of the bacterial cellul1ose-based cosmetics in the last decade.

## 2. Results

**Figure 1:**
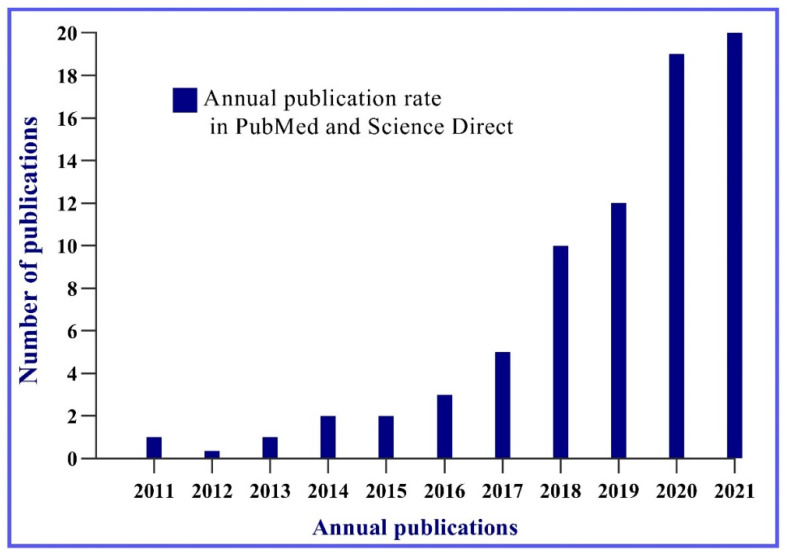
Graphical representation of the perspective of publications on the use of bacterial cellulose in cosmetics in the last decade.

The survey in the PubMed and Science Direct platforms resulted in a total of 70 articles. In Figure 1, it is possible to observe that over ten years, there was a significant increase in publications related to bacterial cellulose and cosmetics. Between the years 2011 and 2015, six articles were published. In 2017, 2018, and 2019, it is possible to visualize an ascendancy in the studies related to the topic with a slight increase in publications. However, in the last five years, the number of publications grew significantly.

In the last two years—2020 and 2021—the greatest growth was observed, with an average of twelve to thirteen articles per year. This is still a relatively small average, but it allows to visualize the scientific prospect around bacterial cellulose in cosmetics, indicating an upward growth trend as illustrated in Figure 1.

**Figure 2:**
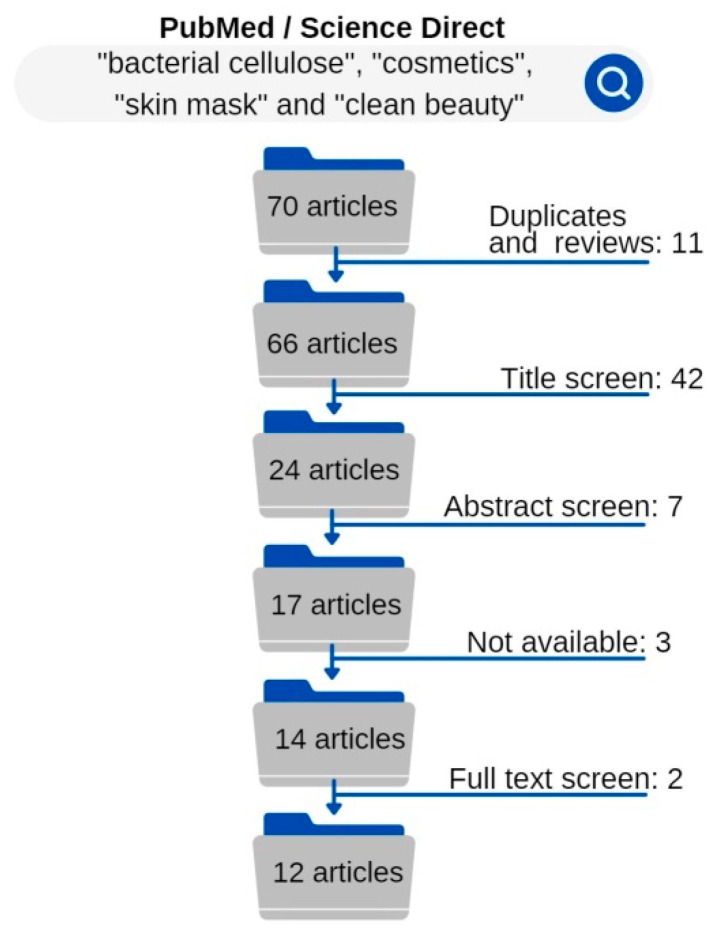
Exclusion criteria delineation.

Figure 2 shows that after the searches, the 70 articles went through the exclusion criteria method. After the first step of exclusion criteria for duplicate studies and reviews, 11 articles were excluded. The titles of the remaining 59 articles were read and evaluated. Then, another 42 articles were excluded because their titles did not reference what was being sought.

Subsequently, the abstracts of 24 articles were read and the abstracts that did not fit inclusion criteria of application of BC in cosmetics were excluded, leaving 17. From these 17, 3 articles were not available for reading. A full reading of 14 studies was then performed and 12 were within all the criteria for data collection.

Table 1 presents the main results. Analytical studies are here described as experimental ones, with direct measurements of chemical compounds. In vitro studies are those conducted outside a living organism, usually on cells kept alive and growing outside their original tissue. Ex vivo studies are performed on extracted tissues or organs. In vivo studies are performed on animals, usually rodents, also called pre-clinical tests. After the pre-clinical studies are performed, the clinical study is conducted, in which the substances are tested in humans.

There is an emerging increase in studies related to BC when applied in cosmetics or in the development of new formulations. Although this review was limited to specific keywords and approaches during the searches, many studies of BC applications in various areas were found, also indicating its innovative and versatile potential.

## 3. Discussion

Among the studies found, another application of BC was described, by LI et al., 2015 [21], as a biomaterial of BC and hyaluronic acid with healing potential for severe skin injury. In this study, BC was tested in vitro on human fibroblast cells, showing no cytotoxicity, decreasing wound healing time, and improving tissue repair performance.

Recently, in 2021, Singh et al. [22], produced mucoadhesive BC for oral applications to protect tissue while healing. The mucoadhesive BC was tested ex vivo and indicated that the product withstood 7 days in a submerged aqueous environment under constant shear stress. The study demonstrated that the mucoadhesive BC has potential for new treatments for diseases and injuries in the oral cavity.

In the search for studies for this review of the application of BC in cosmetics, it was found that in 2011, Amnuaikit et al. [13], conducted a clinical study with 30 healthy Thai volunteers, aged between 21 and 40 years old. Among them, there were 21 women and 9 men. The masks used in this study had been produced from the fermentation of the bacteria *Acetobacter xilinum* in rice substrate. The masks were then steam-sterilized at 121 °C. In an attempt to test the moisturizing capacity of BC on the skin, the mask was applied to the volunteers for a period of 25 min, and within 5 min from the removal of the product, the levels of moisture and flaking, elasticity, and other parameters were evaluated. All volunteers participated until the end of the study because there was no skin irritation after 24 h of BC application. The results showed that BC significantly increased skin hydration after a single application for all volunteers. This can be considered one of the first scientific records of the potential of BC in human skin applications.

Almeida et al., 2013 [14], tested BC as a possible adhesive material on 15 healthy volunteers, 12 women and 3 men, aged 30–40 years old. The BC membranes in that study were produced using *Gluconacetobacter sacchari* in Hestrin and Shramm (HS) liquid medium, and subsequently washed, bleached, and oven dried at 40 °C for 16 h. After this process, 1% glycerin was incorporated into the BC membranes. Then, the product was applied to the forearm of the volunteers and two visual assessments of the degree of skin irritation were performed after the removal of the BC patches. No high levels of skin irritation were observed, proving the safety of BC as a cosmetic product.

In addition to positive results in its pure state as a potential moisturizer, BC was studied as a carrier and distributor of cosmetic pharmaceutical drugs by Numata et al. in 2015 [15]. In this study, BC gels were biosynthesized from the fermentation of *Gluconacetobacter xylinus* in liquid HS medium for 3 weeks under static conditions. Subsequently, the gels were purified in tap water for 2 days, deproteinized in 0.5% (*w*/*w*) NaOH solution, neutralized in 0.5% (*w*/*w*) acetic acid solution, and washed with distilled water. The incorporation process of the BC gels was performed by immersion in the retinol-loaded nanoparticle suspension for 24 h at room temperature. In in vitro release assays, BC also proved to be a great tool in the cosmetic industry.

In 2017, BC membranes produced from *Gluconacetobacter xylinum* bacteria in liquid medium (HS) were incorporated with ZnO and tested in 20 BALB/c mice to assess tissue regeneration capacity. For this assay, the produced BC membranes were processed by washing with distilled water followed by autoclaving in 3 M NaOH solution at 120 °C for 15 min to break up and dissolve remnants of organic matter. Next, the BC membranes were incorporated by dipping bacterial cellulose films into ZnO nanoparticle suspension and mixed in an incubator with stirring at 50 °C and 150 rpm for 24 h.

In this in vivo study, burn wounds were induced in animals. In one group, the wounds were treated with BC-ZnO nanocomposites and in the other one, only with BC. As a result, optimal performance of BC for potential tissue healing along with antimicrobial activity was obtained according to Khalid et al. [16]. These BC composites could be suggested as potential skin wound healing material after clinical evaluation.

In another study, the BC produced by *Gluconacetobacter xylinum* in liquid medium HS was incorporated with two formulations: one with an association of oat extract, rosemary, calendula, and hydroviton; and another one with propolis extract. The BCs were immersed in a tray and the cosmetic actives were homogeneously distributed on its surface. The trays were agitated for 30 min. The membranes were turned every 5 min to provide a good distribution of the actives. As a result, two types of mask were produced and tested on 20 women volunteers, between 20 and 40 years of age, to evaluate the moisturizing action of the extracts. The masks were applied for 20 min for 5 consecutive days. The volunteers did not use any type of cosmetic during the experimental period. The results obtained by Pacheco et al., 2017 [7], indicated that the BC mask was effective in delivering the substances; however, the increase in hydration was only observed in the BC mask with plant extracts.

In 2018, BC was studied by Jantarat et al. [17], as a composite biomaterial that allowed optimal ex vivo release time of piperine. Unlike the other studies, in this one, the membranes were purchased ready-made from a local market in Songkhla, Thailand. For the study, the membranes were ground and mixed with the piperine and curcumin solutions separately, then dried at 60 °C for 2 h in Petri dishes. Then, they were layered one on top of the other. The BC containing curcumin and piperine showed potential for the controlled release of curcumin transdermally, and was shown to facilitate the bioavailability of curcumin.

In 2018, Perugrini [18] tested BC masks purchased from two different suppliers to evaluate the effects of short- and long-term BC use. The study was conducted on 89 healthy women for one month with application three times a week. The short- and long-term effects of the BC masks were analyzed. It was concluded that the shelf life of BC is six months. No side effects were reported from use, again proving the safety of using BC in cosmetics.

And in 2019, Pergurini et al. [19] demonstrated the feasibility and efficacy of BC obtained by incubating *Gluconacetobacter xylinus*. Three different solutions were incorporated into BC. The first one was an anti-aging mascara with Adansonia digitata, Hibiscus sabdariffa flower, Arabic coffee seed, African Kigelia fruit and *Crocus chrysanthus* extracts as well as hydrolyzed hyaluronic acid and Senegal acacia gum.

The second one was a lifting mascara with hydrolyzed Quinoa seed extract and hydrolyzed hyaluronic acid. Lastly, the third solution was a purifying and regenerating mascara with *Portulaca oleracea* and *Chlorella vulgaris* extracts in addition to sodium hyaluronate, kaolin, and magnesium sulfate.

In the tests with volunteers, all three mascaras were applied for one month and the formulations showed excellent results indicating efficacy and safety. The extracts were chosen based on their natural biomedical properties such as antioxidant action, potential to increase skin hydration and elasticity, and activation of regeneration processes, among other benefits found in the literature regarding the chosen actives. Once again, the benefits of BC as an aid in the development of safe and natural cosmetics were proven.

Chantereau, et al., 2020 [12] studied BC membranes with the size of 2 × 2 cm produced by the *Gluconacetobacter sacchari* strain and incorporated in 1 mL of the ionic liquid loaded solution and vitamin B. The membranes were used as cosmetic adhesive material and showed high thermal stability and ability to replace the use of plasticizers. The incorporated BC also increased the bioavailability of vitamin B, aided skin hydration, and showed no cytotoxic activity.

Bacterial nanocellulose produced by *Gluconacetobacter sacchari* has also been studied as a delivery model for hyaluronic acid in patch form with regenerative moisturizing properties [8]. The model was tested in vitro on HaCaT cells to assess cytotoxicity and ex vivo on pig ear skin to assess its permeation ability. The results of the trials were successful and proved to be safe for the application of these adhesives.

In 2020, Malmir et al. [20], used BC produced by the bacterium *Gluconacetobacter xylinus* in HS medium to develop an antibacterial dressing with carbon quantum dioxide titanium nanoparticles (CQD-TiO_2_). To incorporate the nanoparticles into BC, the CQD-TiO_2_ solution was sprayed into wet BC at room temperature. Then, using the heat press machine, the membranes were dehydrated and dried for 3 days at 40 °C. This dressing model was tested in vitro on human L929 cells and CQD-TiO_2_ loaded into BC was non-cytotoxic and effective in skin wound healing. To evaluate the antibacterial ability, a minimum inhibitory concentration test was performed on *Staphylococcus aureus*, proving the antibacterial activity.

## 4. Experimental Articles

**Figure 3:**
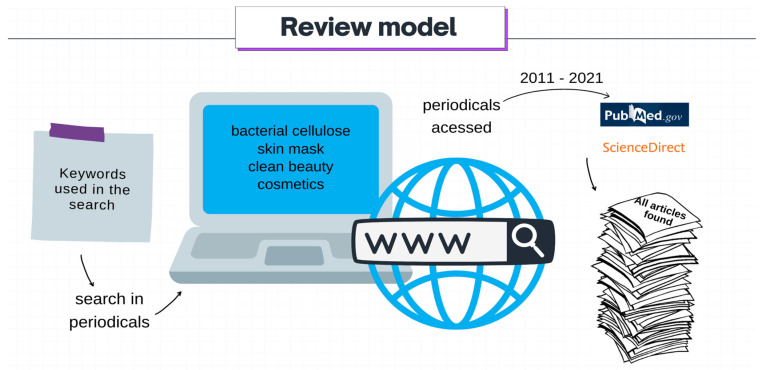
Diagram used to define and limit the search for studies based on key words.

Figure 3 shows how the systematic electronic research was conducted on two search platforms (Science Direct and PubMed) throughout the month of January 2022. The literature search strategy combined the descriptors “bacterial cellulose”, “cosmetics”, “skin mask”, and “clean beauty”. The descriptors were chosen to be specific enough not to cover too much irrelevant literature. Furthermore, the descriptors were selected based on the “Medical Subject Headings (MeSH)”. The research was conducted over a ten-year period, between 2011 and 2021.

Searches were restricted by language (only English language). All original articles, related to development of products or new approaches, were considered for this review.

First, before the articles were evaluated, the year of each publication was collected to emphasize the prospect of growth of bacterial cellulose research in cosmetics. The software GraphPad Prism 8 was used to demonstrate this perspective.

**Figure 4:**
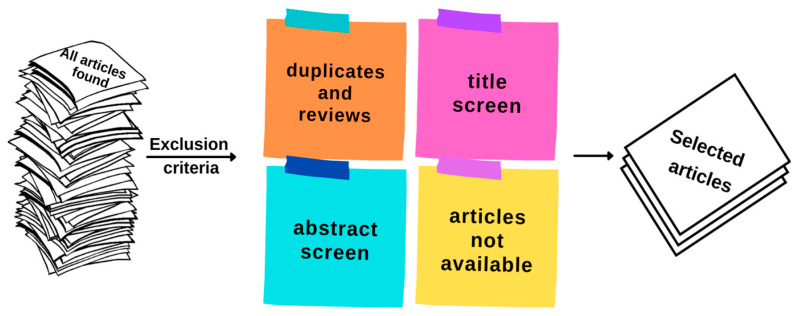
Exclusion criteria to define the studies that were used.

After that, one reviewer independently accessed each article that was included in this review. Two other reviewers assisted in meticulous reading and confirmed the accuracy of the data that was included and excluded. Figure 4 shows there was a careful selection of articles based on the exclusion criteria, which defined the corresponding articles to be investigated.

In the selection, duplicate studies and review articles were excluded, as well as studies that did not specifically address the subject, and studies that did not use bacterial cellulose as primary material. After this first selection, the titles were screened by abstracts. Studies that did not correspond to the criteria of the searched data were excluded.

For the complete review of the data sought, the full texts of the pre-selected articles were obtained, but not all of them were available to collect the necessary and pertaining data. Data were collected based on the application of BC in cosmetics, the type of microorganisms that produced the BC, the type of the study (in vitro, in vivo, and clinical studies), and also the purpose of the product or technology developed in the article. Data were grouped in a descriptive table.

## 5. Conclusions

Although limited in number, the studies on the application of bacterial cellulose in cosmetics used in this review indicate that this is a very promising area for researchers interested in natural cosmetics. In the presented studies, BC shows ideal pharmaco-cosmetic characteristics, such as moisturizing potential, oiliness control, controlled release of substances, biocompatibility with human tissues, and increased bioavailability of certain substances. In addition, it also proves to be safe for skin application because it is a bioproduct that does not harm the consumer.

We believe that cosmetics developed from the green technology of bacterial cellulose are committed to innovation, sustainable practices, circular economy and conscious beauty replacing unhealthy and dangerous substances. Bacterial cellulose may be a potential ally for the consumer and environment due to its natural origins.

## Figures and Tables

**Table 1:** Data demonstration collected in the studies on bacterial cellulose in cosmetics selected by the aforementioned exclusion criteria.

Results	Microorganism	Study	Reference
The BC mask significantly increased the skin moisture after a single application.	*Acetobacter xylinum*	Clinical: 30 volunteers	Amnuaikit, et al., 2011 [13]
A tolerance under occlusion, after a single application, reinforced the putative interest of BC membranes as support for drug topical delivery.	*Gluconacetobacter sacchari*	Clinical: 15 volunteers	Almeida, et al., 2013 [14]
The combination of BC gel and nanoparticles is a slow-release system that may be useful in the cosmetic and biomedical fields for skin treatment and preparation.	*Gluconacetobacter xylinus*	Analytical	Numata, et al., 2015 [15]
The BC was incorporated with two formulations, one with plant extracts and the other one with propolis extract, so two types of masks were produced. The BC was effective in delivering the two subtances; however, the increase in hydration was only observed in the masks with plant extract.	*Gluconacetobacter xylinus*	Clinical: 20 volunteers	Pacheco, et al., 2017 [7]
BC impregnation with ZnO nanoparticles increased considerable the antimicrobial activity of BC. Furthermore, the synthesized composite enhanced wound healing and tissue regeneration activity.	*Gluconacetobacter xylinum*	in vivo: Balb/C mice	Khalid, et al., 2017 [16]
BC was designed with the lower layer composed of piperine and the upper layer composed of curcumin. This form of system allowed the release of piperine from the system and interacted with the skin structure before curcumin was released through the skin.	*Gluconacetobacter xylinus*	ex vivo: pig ears skin	Jantarat, et al., 2018 [17]
Non-destructive assay application for evaluating the stability and safety of BC in humans.	*Gluconacetobacter xylinus*	Clinical: 89 volunteers	Perugrini, et al., 2018 [18]
Application of BC masks for 1 month improved the firmness and elasticity of the skin and assisted in cell renewal.	*Gluconacetobacter xylinus*	Clinical: 69 volunteers	Perugini, et al., 2019 [19]
Incorporation of vitamin B-based ionic liquids into BC for their use in skin care. The incorporated BC increased the bioavailability of vitamin B, helped with skin hydration, and showed no cytotoxic activity.	*Gluconacetobacter sacchari*	in vitro: HaCaT cells	G. Chantereau, et al., 2020 [12]
BC was used as a carrier for hyaluronic acid and formulated as a patch with moisturizing and regenerative properties. The developed patch proved to be safe and effective.	*Gluconacetobacter sacchari*	in vitro: HaCaT ex vivo: pig ears skin	Fonseca, et al., 2020 [8]
An antibacterial dressing was produced with BC as a carrier for titanium quantum carbon dioxide nanoparticles (CQD-TiO_2_). The model was tested in vitro and proved to be non-cytotoxic and effective in skin wound healing.	*Gluconoacetobacter xylinum*	in vitro: human L929 cells	Malmir et al., 2020 [20]
BC was used as a carrier matrix for the Epilobium angustifolium extract with the intention of a controlled release of the plant extract for local delivery of antioxidants to the skin. The results indicated increased antioxidant properties and full release of the extract.	*Komagataeibacter xylinus*	in vitro: L929 mouse ex vivo: pig skin	Nowak, et al., 2021 [10]

## Data Availability

Not applicable.
